# Flexural Strength of Different Monolithic Computer-Assisted Design and Computer-Assisted Manufacturing Ceramic Materials Upon Accelerated Aging

**DOI:** 10.1055/s-0044-1785185

**Published:** 2024-05-14

**Authors:** Niwut Juntavee, Apa Juntavee, Orapun Chansathien, Natcha Prasertcharoensuk, Boonsita Leesuraplanon

**Affiliations:** 1 Department of Prosthodontics, Faculty of Dentistry, Khon Kaen University, Khon Kaen, Thailand; 2 Division of Pediatric Dentistry, Department of Preventive Dentistry, Faculty of Dentistry, Khon Kaen University, Khon Kaen, Thailand; 3 Division of Biomaterials and Prosthodontics Research, Faculty of Dentistry, Khon Kaen University, Khon Kaen, Thailand

**Keywords:** aging, flexural strength, lithium disilicate, monolithic zirconia, zirconia-reinforced lithium silicate

## Abstract

**Objectives** The durability of ceramic is crucial, which is probably influenced by aging. This study evaluated the effect of aging on flexural strength of different ceramics.

**Materials and Methods** One-hundred twenty ceramic discs (Ø 12 mm, 1.5 mm thickness) were prepared from zirconia-reinforced lithium silicate (ZLS, C), lithium disilicate (LS _2_ , E), precolored yttria-stabilized tetragonal zirconia polycrystalline (Y-TZP, Ip), and customized color Y-TZP (Ic). Samples were randomly divided into two groups for accelerated aging (A) between 5 and 55°C water baths, 30-second immersing time each, for 10,000 cycles, and nonaged group (N), serving as control. Biaxial flexural strength (σ) was evaluated utilizing the piston-on-three-balls at 0.5 mm/min speed. Analysis of variance and Tukey comparisons were determined for significant differences ( *α* *=* *0.05* ). Weibull analysis was applied for survival probability, Weibull modulus (m), and characteristic strength (σ _o_ ). Microstructures were evaluated with scanning electron microscopy and X-ray diffraction (XRD).

**Results** The highest σ and σ _o_ were seen for IcN, followed by IcA, IpN, IpA, EN, CA, CN, and EA, respectively. CN showed the highest m, while EA showed the lowest m. Significant differences of σ for each ceramic were indicated ( *p <* 0.05). Aging caused a significant difference in σ ( *p <* 0.05). XRD showed t→m phase transformation of Ip and Ic after aging.

**Conclusion** Aging affected strength of ceramics. Comparable strength between LS _2_ and ZLS was evidenced, but both were less strength than Y-TZP either aging or non-aging. Comparable strength between precolored Y-TZP and customized color Y-TZP was indicated. Better resisting aging deterioration of Y-TZP than LS _2_ and ZLS is suggested for fabrication restorative reconstruction.

## Introduction

Dental ceramics have been used in dental practice for many years to restore the natural characteristics and function of dental structures. Recently, dental ceramic restoration has become popular and plays an important role in the contemporary practice of restorative dentistry as a result of modern technology of computer-assisted design and computer-assisted manufacturing (CAD-CAM), instruments, materials, and treatment techniques that are capable of providing a natural esthetic restoration within an appropriated period of treatment. 1 Restorative dentistry materials must possess proper mechanical and aesthetic properties and be resistant to fracture. Improvements in materials are needed for the strength with proper application in the load-bearing area. The demands of patients regarding esthetics, as well as the development of technology, have convinced most clinicians to use materials that possess extraordinary optical properties to replicate the color of natural dentition and the ability to withstand heavy occlusal force. 2 As such, ceramics are advocated as restorative materials in conjunction with their biocompatibility. 3 Over the last three decades, there has been a rapid expansion of ceramic use, leading to a variety of new ceramic materials. Lithium disilicate glass ceramics (LS _2_ ) are the most widely used ceramic materials for fixed dental prostheses due to their good mechanical properties and their extraordinary translucency, which is ideal for restoration in the aesthetic zone. 4 5 The LS _2_ is a material in the glass-matrix ceramic group of dental ceramics. It looks like natural teeth because of their excellent optical properties. Its microstructure consists of elongated LS _2_ crystals embedded in a glassy matrix. It exhibits a translucency and aesthetic appearance superior to that of zirconia due to its glassy matrix. 6 The optical properties of this material depend on the crystal size and differences in the refractive index of the glass matrix and the crystalline structures. 5 This material is not only used for fabricating laminate veneer, single and multiunit fixed partial dentures in the anterior region but also used as monolithic fixed partial dentures in the posterior region. 7 To increase the strength of LS _2_ glass-ceramics, more zirconia was added, then called zirconia-reinforced lithium silicate (ZLS) glass-ceramics, and this was advocated for use in the heavier occlusal force area over the original LS _2_ glass-ceramics. ZLS has been developed by combining the positive characteristics of zirconia and LS _2_ glass. 8 . The high strength of ZLS is provided by adding approximately 10% zirconia content to a glass matrix. 5 Its microstructure contains tetragonal zirconia grains dissolved in a homogeneous glassy matrix that consists of the very fine grain of lithium metasilicates and lithium disilicates. 9 This material not only supported the aesthetic demands of the patients but also contained the strength to resist occlusal force, as in the posterior restoration.

One of the most recent innovative ceramics is zirconia. Zirconia comprises an entirely polycrystalline structure that presents extraordinary mechanical strength, fracture toughness, chemical resistance, and dimensional stability and possesses high biocompatibility. 2 10 Zirconia has been increasingly used for manufacturing various restorations, including fixed dental prostheses and implant restorations. 11 There are three phases of crystal structure in zirconia: monoclinic (m), tetragonal (t), and cubic (c) phase. The m phase is detectable at room temperature, and the t phase can be observed when the temperature reaches 1170°C. The t phase can change to the c phase at 2370°C. The c phase remains unaltered until the melting point is reached at 2680°C. The t and/or c phases of zirconia can remain stable at room temperature by adding oxide stabilizers such as yttrium oxide (Y _2_ O _3_ ). 10 12 External stimulation, such as humidity, heat, and stress, can cause the transition of zirconia from the t→m phase, which leads the microstructure to expand by 4 to 5%. 13 This volumetric shift leads to “transformation toughening” that is what gives stabilized zirconia its exceptional strength by inducing compressive stress to withstand the spread of cracks. 4 14 In contrast, the opacity of yttria-stabilized tetragonal zirconia polycrystalline (Y-TZP) has been taken into account. It was manufactured primarily to produce substructures for porcelain veneering to achieve greater translucency. However, there have been numerous reports of delaminating and chipping of porcelain overlays. 10 15 16 17 Monolithic zirconia has been developed to solve this problem and also improve esthetics. 18 It was produced in the form of precolored and customized color monolithic Y-TZP. However, its translucency and color stability are still inferior to LS _2_ . 19 20

Ceramics can respond to dentist and patient expectations due to their esthetics, good mechanical properties, and biocompatibility, which are very important for clinical selection. The compressive strength of ceramic is high, but it is brittle and cannot withstand high tensile strength. The performance of dental ceramic restorations was assessed with an emphasis on durability. The flexural strength test is a crucial and reliable technique that is frequently used to assess the resilience of ceramic materials. A high flexural strength restoration indicates less chance of fracture. 21 The Weibull modulus is commonly used to describe different aspects of structural variability and the distribution of faults of brittle materials. Structural reliability is directly proportional to the Weibull modulus (m value). Most dental ceramics show m values between 5 and 15. 22 According to ISO 6872:2015, the characteristic strength (σ _o_ ) is defined as the strength present at a 63.2% probability of failure for specific loading configurations and test specimens. The ability to withstand failure is proportional to the characteristic strength. 23 The flexural strength of ceramic can be measured with two techniques: uniaxial flexural strength tests such as three-point bending or four-point bending and biaxial flexural strength tests such as piston-on-three-balls or ball-on-ring or ring-on-ring tests 24 that depend on shape and size of specimens and testing procedure. 25 In all cases, the static load is applied until the fracture of the specimen.

Aging is an autodegradation process for a certain period, which is related to the chemical and mechanical properties of materials. Long-term use of materials is related to the performance threshold of materials to endure stress during their function in oral environments. 2 26 27 The methods to evaluate the flexural strength of the ceramic materials immediately after the sintering process limit the ability to determine the clinical behavior of the materials. In addition, the determination of material failure without the aging process is probably not respected with the nature of the durable behavior of the material. Thermocycling is the popular method to accelerate the artificial aging of the specimens in the experimental study for evaluating the long-term clinical success of ceramic restoration. 28 The material aging through the thermocycle aging process induces fatigue in the material and speeds up the deteriorated effect on the material. Artificial aging through the thermocycling process probably accelerates the hydrolysis phenomenon at the interface components and destroys disintegrated products. Thermocycle aging eventually induces an increasing difference in the thermal expansion coefficient between ceramic materials and natural tooth dentine, probably leading to a breakdown of restorative ceramic. Some studies conclude that the aging of ceramic materials affects their flexural strength. 2 10 26 There is still a controversial effect of aging on the flexural strength of ZLS, LS _2_ , and Y-TZP. 12 15 Likewise, no study until recent has investigated the distinction in strength concerning precolored and customized color monolithic Y-TZP. As such, the purpose of this study was to compare the flexural strength between aged and nonaged ZLS, LS _2_ , precolored, and customized color monolithic Y-TZP and apply them in clinical use appropriately. The null hypothesis was that no significant difference in the flexural strength of various ceramic materials upon accelerated aging.

## Materials and Methods

An experimental study was conducted to investigate the effects of artificial aging on the biaxial flexural strength of four monolithic ceramics. The sample size for the *in vitro* study was estimated using the G*power 3.1 software (Heinrich-Heine-Universität, Düsseldorf, Germany) based on the statistical data from the former study 13 using tests powers = 0.9, and α error = 0.05 as shown in Eq. (1) .



Where *Z*_α =_ standard normal deviation = 1.96 (α error = 0.05), *Z*_β =_ standard normal deviation = 1.28 (β error = 0.1), *µ*_1_ – *µ*_2_ = mean difference between experimental group = 0.8 and *s* = standard deviation ( *s*_1_ =2.3, *s*_2_ = 1.5)

The number of sample sizes based on this calculation was 15 samples per group used for this experiment.

### Ceramic Specimen Preparation

Four monolithic ceramic blocks of shade A3 as presented in Table 1 —zirconia-reinforced lithium silicate glass (ZLS, C, Celtra Duo, Dentsply DeTrey, Konstanz, Germany), lithium disilicate glass (LS _2_ , E, IPS E.max CAD, Ivoclar Vivadent, Schaan, Liechtenstein), pre-colored monolithic zirconia (Ip, inCoris TZI, Sirona Dental Systems, Bensheim, Germany). and customized color monolithic zirconia (Ic, inCoris TZI, Sirona Dental Systems)—were sectioned into the disc shapes ( *n* = 30 for each type) using diamond coating blade (Mecatome T180, Presi, Eybens, France) with 0.01 mm accuracy. The disc samples were grounded with silicon carbide abrasive # 200, 400, 600, 800, 1000, 1200, and 2000, respectively, and then polished with a 1 µm diamond suspension using a polishing machine (Ecomet3 polisher, Beuhler, Lake Bluff, Illinois, United States) to achieve the desired dimension (12 mm in diameter (Ø) and 1.5 mm thickness). The inCoris TZI disc samples were then cut to oversized dimensions (15 mm in Ø and 1.88 mm thickness) to compensate for the 20% shrinkage after sintering. 19 All samples were cleaned in distilled water for 15 minutes using an ultrasonic cleaner (Vitasonic II, Vita Zahnfabrik, Germany), and then allowed to dry at room temperature for 60 minutes. The E-specimen was glazed with IPS E.max CAD Crystall/GlazeSpray (Ivoclar Vivadent) and sintered according to the manufacturer's instructions with the ceramic furnace (Programat P310, Ivoclar Vivadent) for both C- and E-specimens. The Ic samples were colored with inCoris TZI coloring liquid (Sirona Dental Systems) using a dipping technique for 5 minutes and sintered with a speed furnace (Infire HTC, Sirona Dental System) following the manufacturer's instructions for both the Ip and Ic samples. The samples were cleaned with distilled water in an ultrasonic cleaner for 15 minutes, and steam cleaned with TouchSteam (Kerr, Orange, California, United States) to eliminate surface debris.

**Table 1:** Material, brand, material abbreviation (Abv.), manufacturers, batch number, and composition (wt%) of ceramic used in this study

Material	Brand	Abv.	Manufacturer	Batch no.	Composition (%Wt)
Zirconia-reinforced lithium silicate glass ceramic	Celtra Duo	C	Dentsply DeTrey, Konstanz, Germany	18028368	SiO _2_ 60%, Li _2_ O 19%, ZrO _2_ < 11%, P _2_ O _5_ < 5%, Al _2_ O _3_ < 2%, Tb _2_ O _3_ < 1%
Lithium disilicate glass ceramics	IPS e.max CAD	E	Ivoclar Vivadent, Schaan, Liechtenstein	S53772	SiO _2_ 57–80%, Li _2_ O 11–19%, K _2_ O < 13%, P _2_ O _5_ < 11%, ZrO _2_ < 8%, ZnO < 8%
Yttria-stabilized tetragonal zirconia polycrystalline monolithic zirconia	Pre-colored inCoris TZI	Ip	Sirona Dental System, Bensheim, Germany	2017202731	ZrO _2_ + HfO _2_ + Y _2_ O _3_ ≥ 99.0%, Y _2_ O _3_ 4.5–6.0%, HfO _2_ ≤ 5%, Al _2_ O _3_ ≤ 0.5%
yttria-stabilized tetragonal zirconia polycrystalline monolithic zirconia	Customized color inCoris TZI	Ic	Sirona Dental System, Bensheim, Germany	2012143120	ZrO _2_ + HfO _2_ + Y _2_ O _3_ ≥ 99.0%, Y _2_ O _3_ 4.5–6.0%, HfO _2_ ≤ 5%, Al _2_ O _3_ ≤ 0.5%

### Aging Process

Each material was randomly classified into two groups ( *n* = 15/group) according to different treatment processes: accelerated aging (A) and nonaging (N) process. Samples from the aged groups were treated in an accelerated aging process in a thermocycling machine (CWB332R-MERL, KMIT'L, Bangkok, Thailand). The thermocycle aging process was performed for 10,000 cycles by soaking the samples in a water bath at 5°C for 30 seconds, resting at room temperature for 20 seconds, then soaking them in a water bath at 55°C for 30 seconds and resting at room temperature for another 20 seconds for each cycle. This approximately corresponds to a year of samples in the oral environment. 29 The nonaged groups were immersed in 37°C of distilled water until testing.

### Flexural Strength Test

**Fig. 1:**
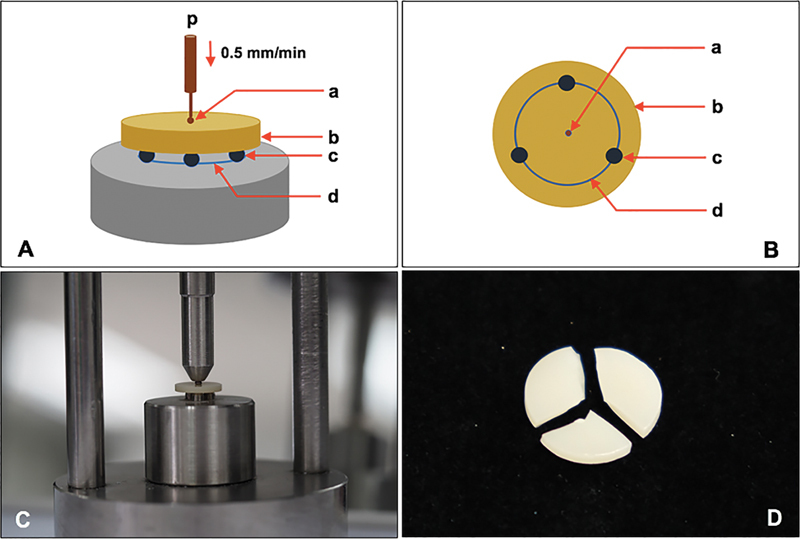
Schematic drawing of piston-on-three-balls of biaxial flexural strength test ( **A** , **B** ). Ceramic disc (b) was placed on three balls (c), which were separately arranged in a circular at 120° apart from each other (d), and loaded vertically (p) with a round end piston (a) at a speed of 0.5 mm/min until fracture ( **C** ). Fracture specimens were further examined microscopically for analysis of fracture ( **D** ).

The specimens were tested for flexural strength on the piston-on-three-ball apparatus ( Fig. 1A ). The testing apparatus comprised three spherical steel balls with a Ø of 4.5 mm, which were arranged in a circular shape with a Ø of 11 mm and separately arranged 120° apart from each other ( Fig. 1B ). The specimens were placed on three spherical balls and pressed with a piston that had a round end Ø of 1.4 mm. Then, the force was induced from a universal testing machine (LR30/k, Lloyd, Leicester, United Kingdom) through the piston for loading at the center of the specimen at a crosshead speed of 0.5 mm/min ( Fig. 1C ). The load was continuously induced until the zirconia fractured ( Fig. 1D ). The load (Newton, N) at failure was recorded and calculated for the biaxial flexural strength (σ, MPa) following Eqs. 2 3 4 . 22







Where *P* is the load at failure (N), *b* is specimen thickness (mm), *υ* is Poisson's ratio (Celtra Duo = 0.25, E.max CAD = 0.23, inCoris = 0.3), *r*_1_ is the radius of supporter (mm), *r*_2_ is the radius of loaded area (mm), and *r*_3_ is the radius of the specimen (mm).

### Microscopic Examination

The surface topography and fracture surface of the samples were labeled and coated with gold-palladium at a current of 10 mA and a vacuum of 130 m-torr for 3 minutes using a sputter coater (K 500X, Emitech, Asford, England), then dried in a desiccator and finally evaluated the microstructures with a scanning electron microscope (SEM, Hitachi S-300N, Osaka, Japan). The crystalline phases of ceramics were determined for the relative amount of microstructure using an X-ray diffractometer (PANalytical, Empyrean, Almelo, The Netherlands). The samples were scanned with copper k-α (Cu Kα) radiation at a diffraction angle (2θ degree) of 20 to 40° with 0.02 step size at every 2-second interval. The phase was analyzed in comparison to the known standard database of the Joint Committee on Powder Diffraction Standards. The ratio of m- to t-phase was determined by peak intensities using the X'Pert Plus software (Philips, Almelo, the Netherlands). The mass fraction of the m phase to the total phase content was calculated from the Garvie-Nicholson formula and further corrected for nonlinearity using the Toraya formula, following Eqs. 5 6 7 . 22







Where *I*_m_ and *I*_t_ are integral intensities of m- and t-phase, respectively. *X*_m_ and *X*_t_ are the Toraya-corrected mass fraction of m- and t-phase, respectively. *C* is theoretically calculated composition-dependent correction, C = 1.32.

### Statistical Analysis

The data of the biaxial flexural strength (σ, MPa) were statistically analyzed for normality using the Shapiro–Wilk test. The mean and standard deviation (SD) of the σ at the point of failure for each group of ceramic materials were calculated, compared, and then further analyzed using bidirectional analysis of variance (ANOVA) in conjunction with multiple posthoc Tukey comparisons using statistical software (SPSS version 22, Chicago, Illinois, United States) to determine significant differences in the flexural strength of ceramic materials with aging. The result was considered statistically significant at the 95% confidence interval (CI). The posthoc Tukey test was used to evaluate the differences between groups. Weibull analysis was used to determine the reliability of the flexural strength and to estimate characteristic strength (σ _o_ ) as well as the Weibull modulus (m) using MS-Excel 2010 (Microsoft, Redmond, Virginia, United States) according to Eq. 8 . The survival graph was created by plotting the reliability against the flexural strength.



Where *P*_f_ (σ) is fracture probability, *σ* is fracture strength, *σ*_0_ is characteristic strength, and *m* is Weibull modulus.

## Results

**Fig. 2:**
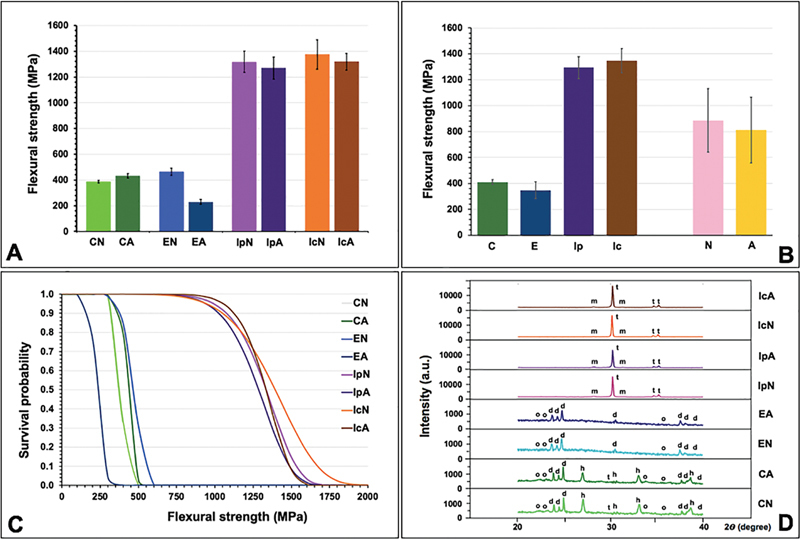
Biaxial flexural strength ( **A** , **B** ), Weibull survival probability ( **C** ), and X-ray diffraction pattern ( **D** ) of Celtra Duo; C, IPS e.max CAD; E, precolored inCoris TZI; Ip, and customized color inCoris TZI; Ic upon aged-; A, and non-aged; N condition. (t: tetragonal phase, m: monoclinic phase, d: lithium disilicate, h: lithium metasilicate, o: lithium orthophosphate).

The mean, SD, 95% CI of σ, σ _o_ , m, the percentage of the grain size distribution, and the relative phase content for each group are shown in Table 2 . The mean ± SD values of σ for each group were ranged from the lowest to the highest: EA (229.6 ± 39.1), CN (386.9 ± 22.8), CA (432.2 ± 36.9), EN (464.4 ± 54.4), IpA (1269.2 ± 167.8), IpN (1317.7 ± 164.2), IcA (1318.9 ± 129.4), and IcN (1375.0 ± 226.8) ( Fig. 2A ).

**Table 2:** Mean, standard deviation (SD), 95% confidential interval (CI) of flexural strength (σ), characteristic strength (σ _0_ ), Weibull modulus (m), relative monolithic (m-), *and* tetragonal (t-) phase content (wt.%), and distribution (%) of fine (0.21–0.48 μm), medium (0.52–0.79 μm), and large (0.83–0.17 μm) grain size of Celtra Duo ( *C* ), IPS e.max CAD (E), pre-colored inCoris TZI (Ip), and customized color inCoris TZI (Ic) upon aged (A), and non-aged (N) condition

Group	*n*	σ (MPa)		σ _o_ (MPa)	m	Relative phase		Grain size distribution (%)		
		Mean ± SD	(95% CI)			m-	t-	Fine	Medium	Large
CN ^a^	15	368.9 ± 22.8	375.4–398.4	397.8	18.1	–	1.00	–	–	–
CA ^a^	15	432.2 ± 36.9	413.5–450.9	449.8	12.3	–	1.00	–	–	–
EN ^a^	15	464.4 ± 54.4	463.9–491.9	490.4	8.7	–	1.00	–	–	–
EA ^b^	15	229.6 ± 39.1	209.8–249.4	247.8	6.1	–	1.00	–	–	–
IpN ^c^	15	1317.7 ± 164.2	1234.7–1400.8	1391.2	8.7	0.12	0.88	20.4	66.7	12.9
IpA ^c^	15	1269.2 ± 167.8	1184.3–1354.2	1343.2	8.3	0.14	0.86	12.6	62.2	25.2
IcN ^d^	15	1375.3 ± 226.8	1260.3–1489.8	1471.4	6.6	0.13	0.87	46.2	43.0	10.8
IcA ^c^	15	1318.9 ± 129.4	1253.5–1384.5	1378.5	10.9	0.16	0.84	30.5	61.0	8.5

NB: Different superscript letters in the group column denoted significant differences between groups ( *p* < 0.05).

The two-way ANOVA indicated a statistically significant difference in the flexural strength of ceramics with the aging process, the type of ceramic, and the interaction effect between the types of ceramic and the aging processes ( *p* *<* 0.05), as shown in Table 3A . Furthermore, one-way ANOVA indicated a statistically significant difference in flexural strength among groups of ceramics upon different aging processes ( *p* *<* 0.05) as indicated in Table 3B . Posthoc Tukey multiple comparisons indicated that Ip and Ic ceramics possessed significantly higher flexural strength than C and E ceramics ( *p* *<* 0.05). However, no significant differences in flexural strength between C and E ceramics ( *p* *>* 0.05) and between Ip and Ic ceramics ( *p* *>* *0.05* ; Fig. 2B and Table 4A ) were indicated. The aging processes revealed a statistically significant effect on the flexural strength of ceramic materials ( *p* *<* 0.05; Fig. 2B and Table 4A ). Posthoc Tukey multiple comparisons indicated significant differences in flexural strength between different monolithic ceramic materials during the aging process, except for the CN-EN, CN-CA, EN-CA, IpN-IpA, IcN-IcA, and IpA-IcA groups ( Fig. 2A and Table 4B ). Weibull analysis of the reliability of flexural strength for different ceramic materials upon aging processes indicated the “m” varied among groups and indicated their relative survival probability of the materials upon flexural strength ( Table 2 and Fig. 2C ). Weibull modulus from the lowest to the highest values was EA (6.1), IcN (6.6), IpA (8.3), IpN (8.7), EN (8.7), IcA (10.9), CA (12.3), and CN (18.1), respectively. The Weibull analysis indicated σ _o_ from the lowest to the highest values: EA (247.8), CN (397.8), CA (449.8), EN (490.4), IpA (1343.2), IcA (1378.5), IpN, (1391.2), and IcN (1471.4), respectively.

**Table 3:** ANOVA of flexural strength of different monolithic ceramic materials upon aging

**A. Two-way ANOVA of flexural strength of monolithic ceramic as the effect of different materials and aging**					
**Source**	**SS**	**df**	**MS**	**F**	***p-*** **Value**
Corrected model	27190335.103	7	3884333.586	223.701	0.001
Intercept	86551267.612	1	86551267.612	4984.533	0.001
Materials	26720143.752	3	8906714.584	512.942	0.001
Treatment	162167.701	1	162167.701	9.339	0.003
Materials* Treatment	308023.649	3	102674.550	5.913	0.001
Error	1944764.476	112	17363.969		
Total	1.157E8	120			
Corrected total	29135099.579	119			
**B. One-way ANOVA of flexural strength of different groups monolithic ceramic material upon aging**					
**Source**	**SS**	**df**	**MS**	**F**	***p-*** **Value**
Corrected model	27190335.103	7	3884333.586	223.701	0.001
Intercept	86551267.612	1	86551267.612	4984.533	0.001
Groups of ceramic	308023.649	3	102674.550	5.913	0.001
Error	1944764.476	112	17363.969		
Total	1.157E8	120			
Corrected total	29135099.579	119			

Abbreviations: ANOVA, analysis of variance; df, degree of freedom; F, F-ratio; MS, mean square; SS, sum of squares.

**Table 4:** Independent *t* -test (A) and posthoc Tukey HSD multiple comparisons (B) of flexural strength of Celtra Duo (C), IPS e.max CAD (E), pre-colored inCoris TZI (Ip), and customized color inCoris TZI (Ic) upon aged (A), and nonaged (N) condition

**A. Posthoc of flexural strength as a function of material types and treatment protocols**								
**Treatment**	**A**	**N**		**Ceramic**	**C**	**E**	**Ip**	**Ic**
A	1	0.001		**C**	1.000	0.261	0.001	0.001
				**E**		1.000	0.001	0.001
N		1.000		**Ip**			1.000	0.398
				**Ic**				1.000
**B. Posthoc of flexural strength among groups of different materials with differed treatment protocol**								
**Groups**	**CN**	**EN**	**IpN**	**IcN**	**CA**	**EA**	**IpA**	**IcA**
CN	1.000	0.742	0.001	0.001	0.981	0.030	0.001	0.001
EN		1.000	0.001	0.001	0.998	0.001	0.001	0.001
IpN			1.000	0.001	0.001	0.001	0.972	1.000
IcN				1.000	0.001	0.001	0.361	0.940
CA					1.000	0.001	0.001	0.001
EA						1.000	0.001	0.001
IpA							1.000	0.968
IcA								1.000

The X-ray diffraction (XRD) microanalysis of the specimens was illustrated as shown in Table 2 and Fig. 2D . The XRD patterns for CN and CA revealed most of the crystal structures of lithium disilicate followed by lithium metasilicate, lithium orthophosphate, and the t-phase of zirconia. The lithium disilicate of CN was observed at the diffraction angle (2θ degree) of 23.88, 24.42, 24.92, 37.71, and 39.92. The CN lithium metasilicate was detected at 2θ degree of 26.99, 30.69, 33.15, and 38.59. The lithium orthophosphate of CN was detected at 2θ degree of 22.26, 23.25, 33.60, and 36.38. The t phase of zirconia for CN was detected at a 2θ degree of 29.73. The lithium disilicate of CA was observed at 2θ degree of 23.85, 24.39, 24.89, 37.66, 38.29, 39.36, and 39.71. The lithium metasilicate of CA was detected at 2θ degree of 26.98, 30.66, 33.04, and 38.60. The lithium orthophosphate of CA was detected at 2θ degree of 22.45, 23.02, 33.78, and 36.43. The t phase of zirconia for CA was detected at a 2θ degree of 29.73. The XRD patterns of EN and EA revealed most of the crystal structure of lithium disilicate with a minor amount of lithium orthophosphate. The lithium disilicate of EN was observed at 2θ degree of 23.76, 24.27, 24.81, 30.28, 30.69, 37.52, 38.18, and 39.18. The lithium orthophosphate of EN was detected at 2θ degree of 22.26, 23.20, 33.83, and 36.33. The lithium disilicate of EA was observed at 2θ degree of 23.71, 24.23, 24.78, 30.62, 37.52, 38.12, and 39.17. The lithium orthophosphate of EA was detected at 2θ degree of 22.083, 23.045, 33.70, and 36.28. The XRD patterns of IpN, IpA, IcN, and IcA revealed most of the crystal structures of the t-phase with a minor amount of the m-phase. The t-phase was observed at 2θ degree of 30.18, 34.66, and 35.21 for IpN; 30.18, 34.67, and 35.21 for IpA; 30.18, 34.59, and 35.13 for IcN; and 30.18, 34.67, and 35.21 for IcA. The m-phase was detected at a 2θ degree of 27.79 and 31.12 for inCoris zirconias. The XRD data of each ceramic corresponded to the crystallographic patterns as indicated in the XRD standard file. The relative concentration of m-phase regarding the total zirconia phase revealed the variation in the amount of the phase transformation from t- to m-phase because of aging, as presented in Table 2 . The relative phase concentrations for the m- and t-phases were 0.12 and 0.88 for IpN, 0.14 and 0.86 for IpA, 0.13 and 0.87 for IcN, 0.16 and 0.84 for IcA. The percentage of phase transformation from t-phase to m-phase was 2.81% for Ip and 3.43% for Ic.

**Fig. 3:**
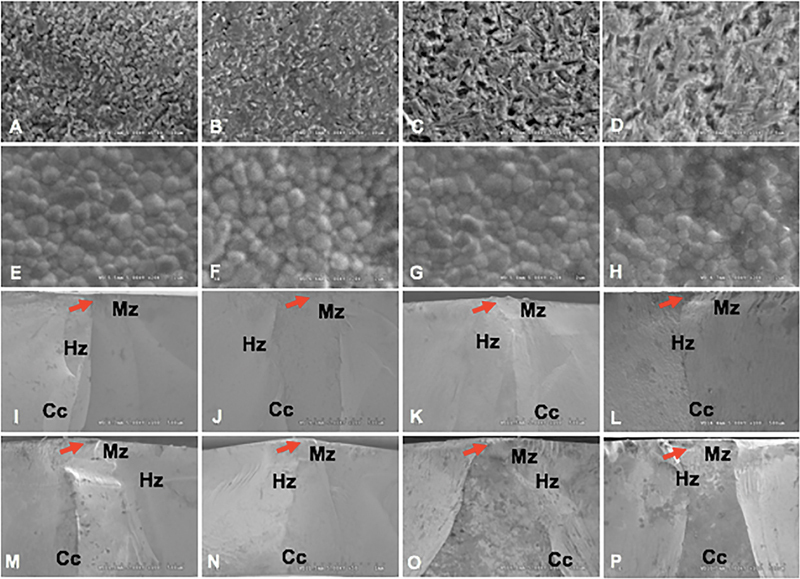
Scanning electron microscopy photomicrographs indicated grain size and grain distribution of monolithic ceramic at X5K magnification (A–H) and fractographic surface at X100 magnification (I–P) indicated fracture origin (red arrow), mist zone (Mz), hackle zone (Hc), and compression curl (Cc) for Celtra DUO (A, B, I, J), IPS e.max CAD (C, D, K, L), precolored inCoris TZI (E, F, M, N), and customized color inCoris TZI (G, H, O, P) upon aged (B, D, F, H, J, L, N, P), and nonaged (A, C, E, G, I, K, M, O) condition.

The SEM micrographs of ceramic materials were exhibited ( Fig. 3A–H ). The Celtra Duo showed a crystal structure of LS _2_ whose size was found in the range of 0.82 to 1.78 μm for CN and 0.55 to 1.64 μm for CA, with a glass matrix. The crystals of the aged group were seen to be more intensive particles than those of the nonaged group. The SEM micrographs for IPS E.max CAD showed the crystal structure of LS _2_ , at a size of 0.72 to 2.25 μm for EN and 1.20 to 2.61 μm for EA, with a glass matrix and some pores. The pore within the aged group showed greater size than the nonaged group. The SEM micrographs for the precolored and customized color inCoris zirconia showed their grains in crystalline patterns that can be divided into the fine grain (0.21–0.48 μm), medium grain (0.52–0.79 μm), and large grain (0.83–1.07 μm). The amount (%) of fine grain, medium grain, and large grain was 20.45, 66.67, 12.88 for IpN; 12.60, 62.20, 25.20 for IpA; 46.15, 43.08, 10.77 for IcN; and 30.51, 61.02, 8.47 for IcA, respectively, as shown in Table 2 . For inCoris ceramics, there were mostly medium grains. Aging resulted in phase transformation and an increase in grain size as illustrated in the increase of large grain in the Ip group and increase in the medium grain in the Ic group. The SEM of the fractographic patterns of each group was presented ( Fig. 3I–P ). The fractographic patterns for E-ceramic indicated that the fracture lines were straight without deviation that originated from the origin and then propagated to the surface of the material, like a catastrophic failure. However, the fracture lines of the remaining groups deviated from the origin to the surface. The fractographic pattern for ceramic C found a few deviations from the fracture path, while the fractographic pattern for Ic and Ip showed more deviation from the fracture path ( Fig. 3M–P ).

## Discussion

The long-term durability of ceramic materials is related to the performance threshold of the material to endure stress during its function in oral environments. Aging has been used to determine the long-term clinical efficacy of dental ceramic materials. The durability of dental ceramics to function is the expected property that can be evaluated by the flexural strength test. A high flexural strength restoration indicates less chance of fracture. The current experiment was performed to determine the biaxial flexural strength of LS _2_ , ZLS, compared with precolored and customized color zirconia after accelerated artificial aging. The results indicated that artificial aging significantly affected the biaxial flexural strength of different types of monolithic ceramic materials. A significant difference in flexural strength was indicated concerning the type of monolithic ceramic materials and their interaction of material and aging process. Therefore, the null hypothesis was rejected for the aging processes and types of ceramic materials and their interactions. The biaxial flexural strength for precolored and custom-color monolithic zirconia was significantly higher than for LS _2_ and ZLS ceramics. However, there was no significant difference in flexural strength between LS _2_ and ZLS as well as between precolored zirconia and customized-colored zirconia. However, LS _2_ before aging indicated slightly higher flexural strength than ZLS for both aged and unaged conditions, which is in agreement with another study. 6

The types of material affect the mechanical properties since they are made up of different components. Monolithic zirconia consists entirely of crystalline content since the crystalline content gives an advantage in terms of strength. Zirconia showed the highest flexural strength compared with others. This was supported by the crack propagation of materials through the occurrence of t- → m-phase transformation. Centra-Duo is mainly composed of crystalline structures: lithium oxide and zirconia, with a glassy silicon dioxide content. Zirconia was added to this material at a level of 8 to 12%, giving so-called ZLS glass ceramic. Although the purpose of adding zirconia to LS _2_ glass-ceramic is to enhance strength through dispersion strengthening, the flexural strength of this material is still lower than the E-groups. This was supported by the XRD, as the low conversion from lithium metasilicate to LS _2_ of C-ceramic, compared with that of E-ceramic, so that C-ceramic showed slightly less flexural strength. Adding more zirconia was shown to hamper crystal growth, as it increased glass viscosity and impeded ion mobility, and thus the rate of solid-state reactions of crystal phase precipitation was reduced. 4

For monolithic zirconia, the result exhibits no significant differences in biaxial flexural strength between precolored and customized color zirconia. This indicated that coloring procedures either precolored or immersion in coloring liquid of monolithic zirconia had no impact on the flexural strength of monolithic zirconia materials. The result of the study was in agreement with other studies. 2 27 However, other studies found a significant impact of the coloring process on the strength of custom zirconia. This is probably related to the variation in the composition and concentration of coloring liquid and the duration of the dipping process. Nevertheless, this study follows the manufacturer's recommendation for a custom coloring procedure for zirconia, which probably leads to no significant difference between precolored and custom-colored zirconia.

Concerning the effect of aging on biaxial flexural strength in the monolithic ceramic materials tested, there was a significant decrease in flexural strength for LS _2_ after aging (from 464.4 ± 54.4 MPa to 229.6 ± 39.1 MPa), but no significant decrease in flexural strength for monolithic zirconia of Y-TZP. Moreover, the study exhibited a slight increase in flexural strength for ZLS after aging (from 386.9 ± 22.8 MPa to 432.2 ± 36.9 MPa), but it was not significantly indicated, which is consistent with the study of Kim et al. 20 The decrease in the flexural strength of LS _2_ is probably related to the deterioration in the microstructures of LS _2_ after aging that is evidenced by the SEM photomicrographs indicating the surface uplifts, microcracks, irregular defect, and enlarging porosities in the material as a result of accelerated aging. These enlarging porosities were the sources of large defects that eventually decreased the flexural strength of the material. Likewise, the difference in the coefficient of thermal expansion (CTE) of the crystalline structure of LS _2_ was greater than the CTE of the glass matrix that caused the development of the tangential compressive stresses around the crystalline structures upon aging and acted as crack deflectors. Nevertheless, if the CTE mismatch between the crystals and the matrix was too high, the crystals could be debonded from the matrix that caused a decrease in flexural strength. Contrariwise, the present study indicated an increase in the flexural strength of ZLS upon aging and significantly higher flexural strength than LS _2_ after aging. This is probably related to the differences in the glassy phase and crystal compositions between LS _2_ and ZLS. The ZLS comprises the fine zirconia dissolved in a glassy matrix. 15 The SEM photomicrograph of ZLS evidenced densely packed crystal structures after accelerated aging. This probably enabled the enhancement of flexural strength of ZLS after aging as evidenced by the fractographic analysis indicating the deviation of fracture path as well as reduction in velocity of crack propagation. For the Y-TZP monolithic zirconia, the study indicated a slight reduction of biaxial flexural strength upon aging, but no significant effect. A previous study reported no significant reduction in the flexural strength of Y-TZP material after aging. 30 This is possibly related to the inherited property of zirconia that is capable of exhibiting low-temperature degradation (LTD) properties. Once the Y-TZP is exposed to the aging process, which was in a humid environment for a long period, the water molecules are capable of penetration into the zirconia structure. The diffusion of water causes tensile stress concentration on the surface of zirconia grains that result in the t→m phase transformation as evidenced in the XRD. According to the XRD patterns for Ip and Ic, the m-phase increased from 0.12 to 0.14% for Ip and increased from 0.13 to 0.16% for Ic after aging, which assuredly confirmed that the LTD phenomenon for Y-TZP ceramics. This phenomenon causes volumetric increases that contribute to surface uplifts and grain pull-out as well as initiating microcracks. Once the t→m phase transformation constantly occurs, the microcracks are enlarged and eventually cause a decrease in the flexural strength of zirconia as supported by another study. 2 Furthermore, this study exhibited the amount of t→m phase transformation was lower for Ip compared with the Ic. This probably assumed that the ability of Ip with aging resistance was higher than that of Ic. However, this study found no significant difference in flexural strength due to accelerated aging in zirconia material; the possible reason was that aging time might be not enough to show a statistically significant difference in flexural strength of zirconia due to aging. This result is consistent with other studies that reported the coloring procedure had a significant effect on the phase transformation but no effect on the flexural strength of the zirconia due to hydrothermal aging. 21 30 The occurrence of LTD is a time-dependent phenomenon, and the rate of phase transformation from t to m increases with increasing aging time. 21 30 Since this study performed the thermocycle aging process for 10,000 cycles, which is comparable to the materials that were placed in the oral environment for 1 year. 29 It was suggested to increase the number of cycles in the thermocycle aging process to 100,000 cycles for a further study to be a more valid evaluation of the long-term performance of ceramic material in an oral environment. Also, this experiment determined flexural strength under the static loading technique. It was recommended to further evaluate the flexural strength under cyclic loading conditions to validate the clinical performance of ceramic material.

## Conclusion

This study indicated that accelerated aging affected the flexural strengths of monolithic ceramic materials. Comparable flexural strength was evidenced between LS _2_ and ZLS, but both had significantly lower flexural strength than Y-TZP either under aging or without aging conditions. Comparable flexural strength was evidenced between precolored and customized color monolithic zirconia, indicating no significant effect of the coloring technique on flexural strength. However, precolored zirconia seems to be capable of resisting aging deterioration better than customized color zirconia. On the contrary, ZLS seems to exhibit better fracture resistance with aging. The study recommended introducing accelerated aging to determine the long-term durability of ceramic materials. Nevertheless, further clinical studies need to be done to get more useful outcomes for the application of ceramic materials in restorative dentistry.

## Clinical Implication

The durability of ceramic plays a critical role in the longstanding success of ceramic restorations. The long-term accomplishment of ceramic is related to the performance threshold of materials to endure stress during function that is comparatively influenced by an autodegradation process of materials for a certain period. Accelerated aging is a useful process to validate the durability of restoration. The monolithic Y-TZP indicated higher durability than LS _2_ and ZLS. Nevertheless, precolored Y-TZP seems to be capable of opposing aging deterioration better than customized Y-TZP and is recommended for the fabrication of ceramic restoration in clinical practice.
